# piRNA-6426 increases DNMT3B-mediated SOAT1 methylation and improves heart failure

**DOI:** 10.18632/aging.203965

**Published:** 2022-03-30

**Authors:** Nier Zhong, Xiting Nong, Jiayu Diao, Guang Yang

**Affiliations:** 1 Department of Cardiology, Shaanxi Provincial People’s Hospital, Xi’an, China; 2 Department of Endocrinology, Xi’an Central Hospital, Xi’an, China

**Keywords:** heart failure, piRNA-6426, DNMT3B, methylation, SOAT1

## Abstract

Purpose: Previous studies found that piRNAs could participate in disease progression by regulating DNA methylation, but there are few reports on their roles in heart failure (HF).

Methods: The level of piRNA-6426 in the venous blood of HF patients and volunteers was detected by RT-qPCR. Hypoxia-induced cardiomyocytes were transfected with lentiviral-mediated piRNA-6426 overexpression vector (LV-piRNA-6426) or together with LV-DNMT3B, and then cell viability and apoptosis, glucose uptake, ROS production, LDH activity and secretion of inflammatory factors were detected. Also, cardiomyocytes were transfected with LV-piRNA-6426, sh-piRNA-6426 or sh-SOAT1, as well as LV-piRNA-6426 or together with LV-DNMT3B or sh-DNMT3B. The interaction between piRNA-6426 and methyltransferase 3B (DNMT3B) was detected with RNA immunoprecipitation (RIP). And the methylation level of sterol o-acyltransferase 1 (SOAT1) and the enrichment of DNMT3B in the SOAT1 promoter were detected with Methylation-specific PCR (MSP) and ChIP assays. Then a HF rat model constructed with coronary artery occlusion method was injected with LV-piRNA-6426, and heart function index and infarcted area of rat heart were detected.

Results: piRNA-6426 expression was decreased in the blood of HF patients. LV-piRNA-6426 transfection increased the enrichment of DNMT3B in SOAT1 promoter, thereby inhibiting the expression level of SOAT1, and decreased hypoxia-induced oxidative stress and inflammation in cardiomyocytes, while sh-piRNA-6426 transfection had the opposite effect. And LV-DNMT3B transfection enhanced the effect of LV-piRNA-6426 transfection on SOAT1 expression and cardiomyocyte dysfunction. Injection of LV-piRNA-6426 significantly inhibited the heart dysfunction of rats.

Conclusions: piRNA-6426 overexpression inhibits hypoxia-induced cardiomyocyte dysfunction and HF by promoting DNMT3B-mediated methylation of SOAT1 promoter.

## INTRODUCTION

The mammalian heart is one of the organs last capable of regeneration, and a variety of factors that cause heart damage could cause heart failure (HF) [1], among them, the long-term hypoxia and ischemia of myocardial cells caused by pressure overload and myocardial obstruction are considered to be the main cause of heart failure [2]. In addition, the heart is also one of the most energy-consuming and oxygen-consuming organs in the body [3]. After myocardial cell injury, mitochondrial dynamics are defective, the uptake of glucose by the cell is hindered, the accumulation of reactive oxygen species in the cell is enhanced, the inflammatory response is intensified, and the expression level of related proteins is inhibited [4, 5]. A study showed that DNA methyltransferase plays a key role in the occurrence and development of cardiovascular diseases [6]. Methyltransferase 3B (DNMT3B) is the most important DNA methyltransferase in the heart of adult mice, and its expression is severely inhibited in the mouse model of HF [7, 8].

The piRNA is a type of small RNA with a length of about 30 nt. It was first discovered in gonadal cells of Drosophila and named piRNA due to its interaction with PIWI family proteins [9]. According to a report, piRNAs secreted by germ cells of drosophila could bind to PIWI protein to promote piRNA recognition and cut transposon mRNAs, which leads to transcriptional inhibition [10]. In mammals, the transcriptional inhibition effect of piRNAs on transposon is mainly achieved by DNA methyltransferase. In this process, piRNAs could guide PIWI protein to silence transposable factors by binding to DNA methyltransferase [11]. A study showed that in multiple myeloma, up-regulation of piRNA-823 could promote the expression of DNMT3B and increase the tumorigenic potential of multiple myeloma stem cells [12]. These studies suggest that methyltransferases may be the target of piRNAs. piRNA-6426 is first detected in the peripheral blood of patients with HF, and the expression level of piRNA-6426 in the peripheral blood of 3 HF patients is lower than that of 3 normal volunteers [13]. While the role of piRNA-6426 in HF is unclear.

Sterol o-acyltransferase 1 (SOAT1) is a key enzyme that converts endoplasmic reticulum cholesterol into cholesterol esters to store lipid droplets [14]. And it is up-regulated in cardiovascular diseases such as atherosclerosis [15] and coronary heart disease [16]. In addition, up-regulation of SOAT1 could further promote the expression level of inflammatory factors [17]. A study found that the methylation level of SOAT1 promoter is lower in patients with coronary heart disease and higher in patients with non-coronary heart disease [16]. DNA methylation refers to the regulation of gene expression without changing the DNA base sequence mediated by DNA methyltransferases [18]. The methylation status of CpG sites near the gene promoter is usually related to transcription activity and gene expression [19]. In recent years, more and more reports showed that the progress of heart failure is related to the methylation level of key genes in cardiomyocytes [20, 21]. However, there is no corresponding report on the effect of SOAT1 promoter methylation level on HF.

In this study, we used hypoxia-induced rat cardiomyocytes to establish HF cell model and the coronary artery occlusion method to establish HF rat model. After overexpression of piRNA-6426, the DNMT3B protein expression level and the SOAT1 promoter methylation level were detected, and a series of cell function indexes and cardiac function indexes were detected to investigate the specific molecular mechanism of piRNA-6426 in regulating HF.

## RESULTS

### piRNA-6426 is down-regulated in the blood of patients with heart failure

**Figure 1:**
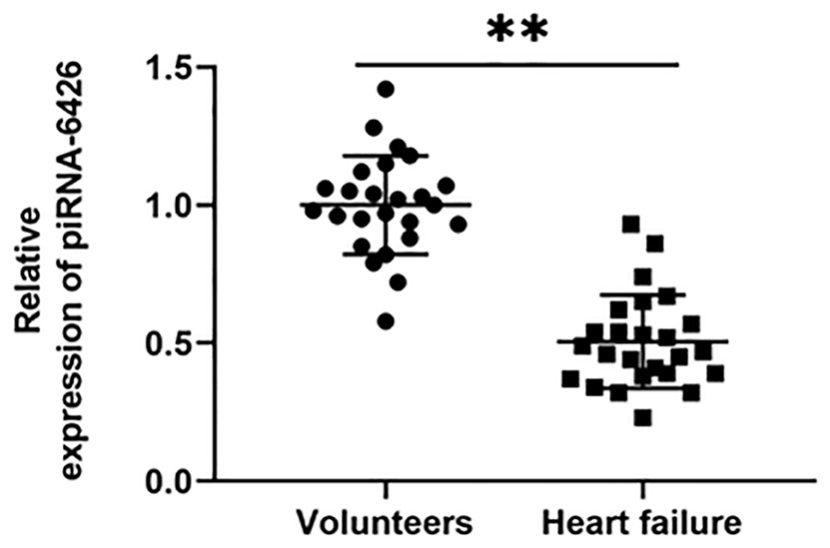
**piRNA-6426 is down-regulated in the blood of patients with heart failure.** After fasting, 5 mL of blood was drawn from the anterior elbow vein of the volunteers and HF patients, RNA was extracted, and the expression of piRNA-6426 was detected by RT-qPCR. Values were expressed as mean ± SEM. **P<0.01, n=25.

We collected blood from 25 normal volunteers and 25 HF patients, and detected the level of piRNA-6426 with PT-qRCR. The results showed that the expression level of piRNA-6426 in the blood of most patients was significantly lower than that of normal samples (Figure 1).

### Overexpression of piRNA-6426 alleviates hypoxia-induced dysfunction of rat cardiomyocytes

**Figure 2:**
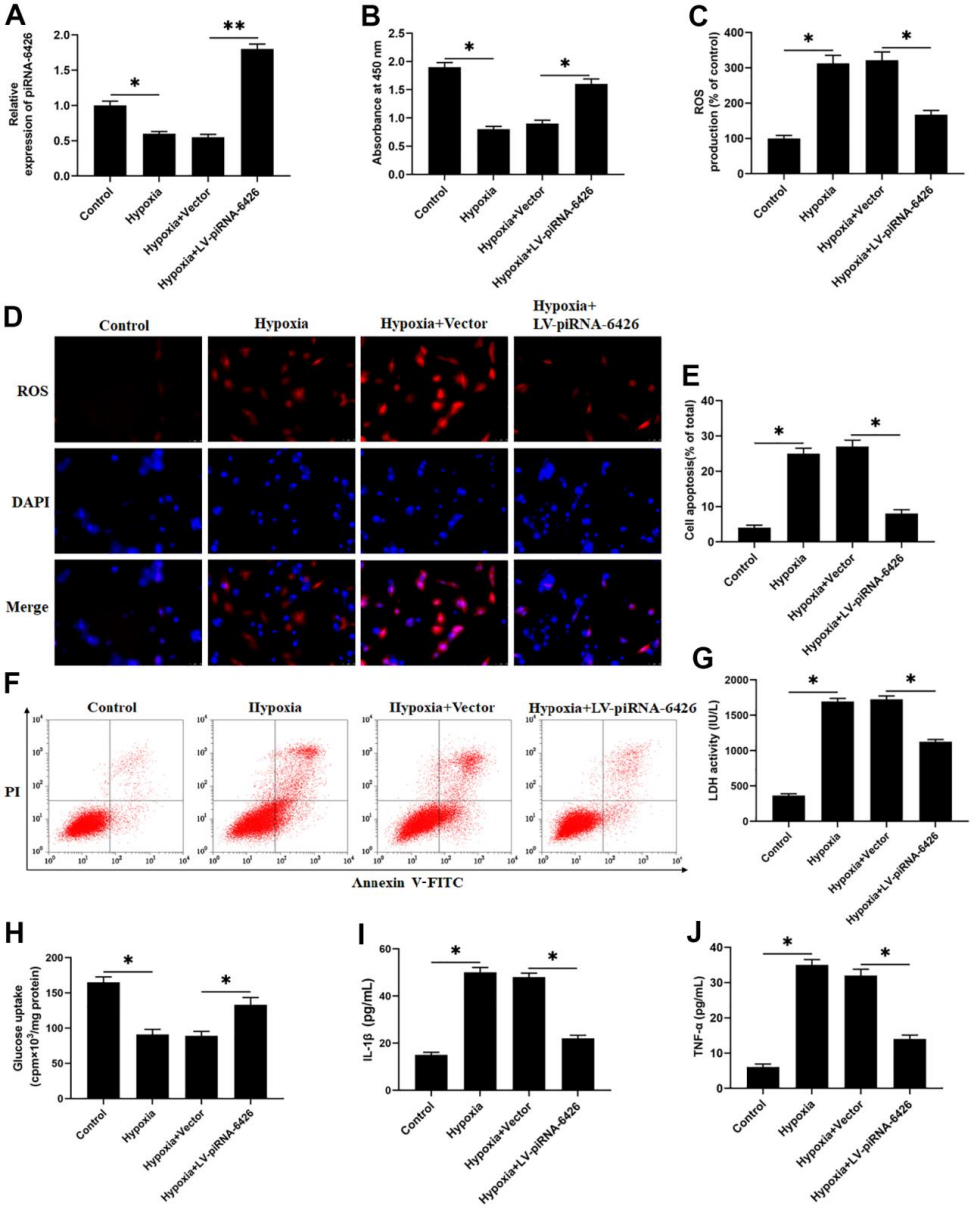
**Overexpression of piRNA-6426 alleviates hypoxia-induced dysfunction of rat cardiomyocytes.** The isolated and cultured rat cardiomyocytes were induced in a hypoxic incubator for 24 hours to establish a HF cell model after 24 h of per-incubated with 25 nM piRNA-6426 overexpression vector. (**A**) The expression of piRNA-6426 in each group were detected by using RT-qPCR. (**B**) MTT assay was used to identify cell viability. (**C**, **D**) The production of reactive oxygen species (ROS) was analyzed with DCFH-DA. (**E**, **F**) Flow cytometry was used to detect cell apoptosis. (**G**) The lactate dehydrogenase (LDH) activity was detected. (**H**) D-(2-3H)-glucose uptake assay was used to perform glucose uptake on fully fused rat cardiomyocytes. (**I**, **J**) ELISA kits were used to detect the secretion of inflammatory cytokines IL-1β and TNF-α. Values were expressed as mean ± SEM. *P<0.05, **P<0.01, n=6.

To verify the effect of piRNA-6426 on cardiomyocyte function, we established a HF cell model by hypoxia-induced rat cardiomyocytes and overexpressed piRNA-6426. We found that compared with the control group, the piRNA-6426 level, cell viability and glucose uptake in hypoxia-induced rat cardiomyocytes were significantly decreased (Figure 2A, 2B, 2H), while cell apoptosis, ROS production, LDH activity and secretion of inflammatory factors IL-1β and TNF-α were significantly increased (Figure 2C–2G, 2I, 2J). Compared with the hypoxia + Vector group, the piRNA-6426 level, cell viability and glucose uptake of cardiomyocytes in piRNA-6426 overexpression group were significantly increased (Figure 2A, 2B, 2H), while cell apoptosis, ROS production, LDH activity and inflammatory factors were significantly decreased (Figure 2C–2G, 2I, 2J).

### piRNA-6426 positively regulates the activity of DNMT3B

**Figure 3:**
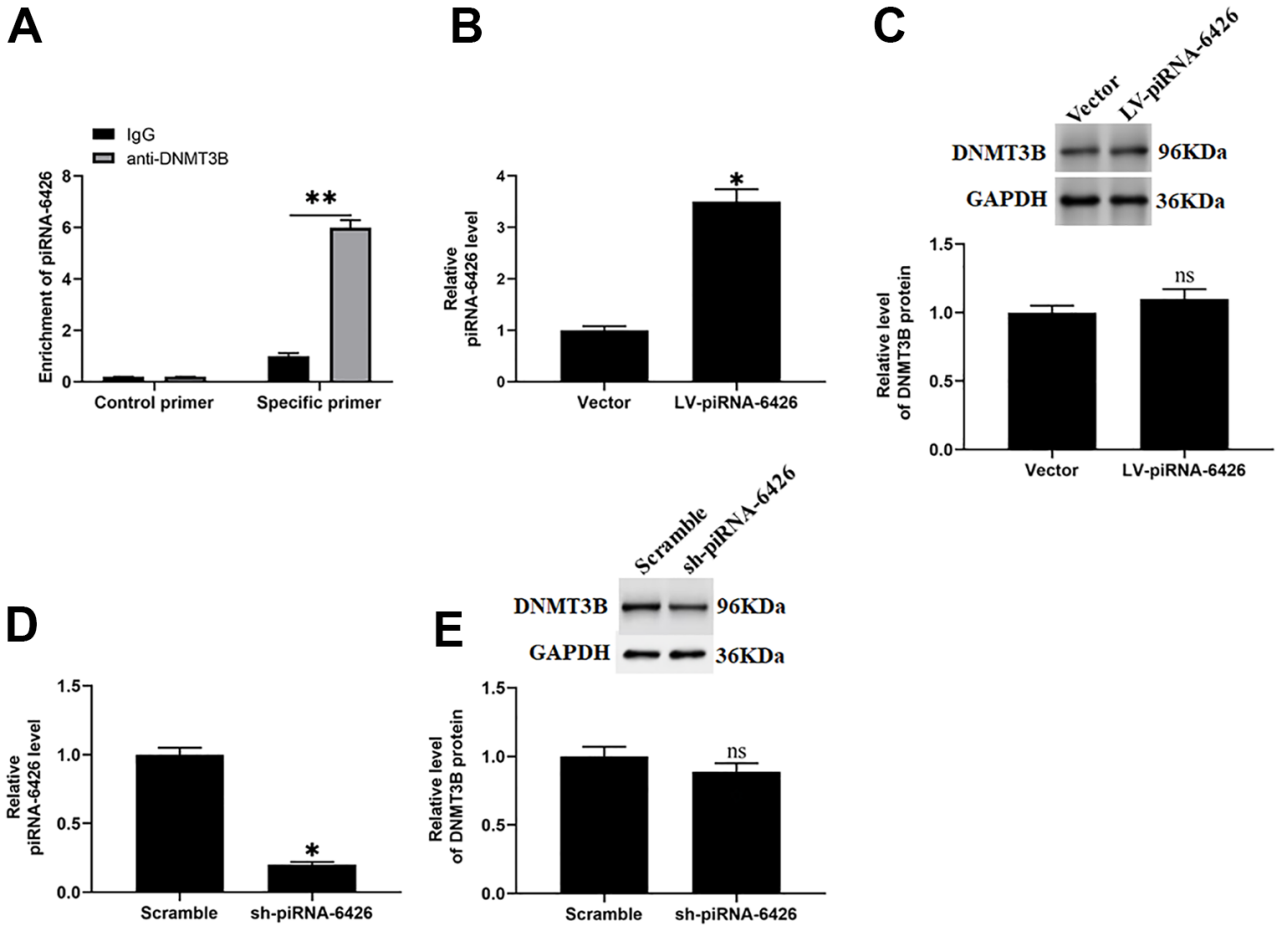
**piRNA-6426 interacts with DNMT3B.** (**A**) Immunoprecipitation method of piRNA-6426 binding to DNMT3B in rat cardiomyocytes. The cells were incubated with LV-piRNA-6426 or sh-piRNA-6426 for 48 h. (**B**) RT-qPCR assay was used to detect the expression of piRNA-6426 in cardiomyocytes incubated with LV-piRNA-6426 or empty vector. (**C**) Western blotting was used to detect the expression level of DNMT3B protein LV-piRNA-6426 in cardiomyocytes incubated with LV-piRNA-6426 or empty vector. (**D**) RT-qPCR assay was used to detect the expression of piRNA-6426 in cardiomyocytes incubated with sh-piRNA-6426 or scrambled shRNA. (**E**) Western blotting was used to detect the expression level of DNMT3B protein LV-piRNA-6426 in cardiomyocytes incubated with sh-piRNA-6426 or scrambled shRNA. Values were expressed as mean ± SEM. ns *P*>0.05, *P<0.05, **P<0.01, n=6.

It is reported that DNMT3B gene knockout mice have severe cardiac dysfunction, suggesting that DNMT3B may be involved in the progression of HF [7]. And piRNAs may co-regulate disease progression with the DNA methyltransferase [22]. In this study, to verify the interaction between piRNA-6426 and DNMT3B in HF, we immunoprecipitated the methyltransferase DNMT3B in rat cardiomyocytes and verified the interaction between piRNA-6426 and DNMT3B through overexpression and interference experiments. As we expected, compared with non-specific control primers, piRNA-6426 enrichment was found in the DNMT3B antibody immunoprecipitated protein-RNA complex of rat cardiomyocytes by using piRNA-6426 specific primers, while no piRNA-6426 enrichment was found in IgG antibody immunoprecipitated protein-RNA complex (Figure 3A). Overexpression of piRNA-6426 increased the expression level of piRNA-6426 (Figure 3B), while piRNA-6426 interference had the opposite effect (Figure 3D). Simultaneously, overexpression and interference of piRNA-6426 had no prominent effect on the expression level of DNMT3B protein (Figure 3C, 3E).

### piRNA-6426 regulates the methylation of SOAT1 CPG island

**Figure 4:**
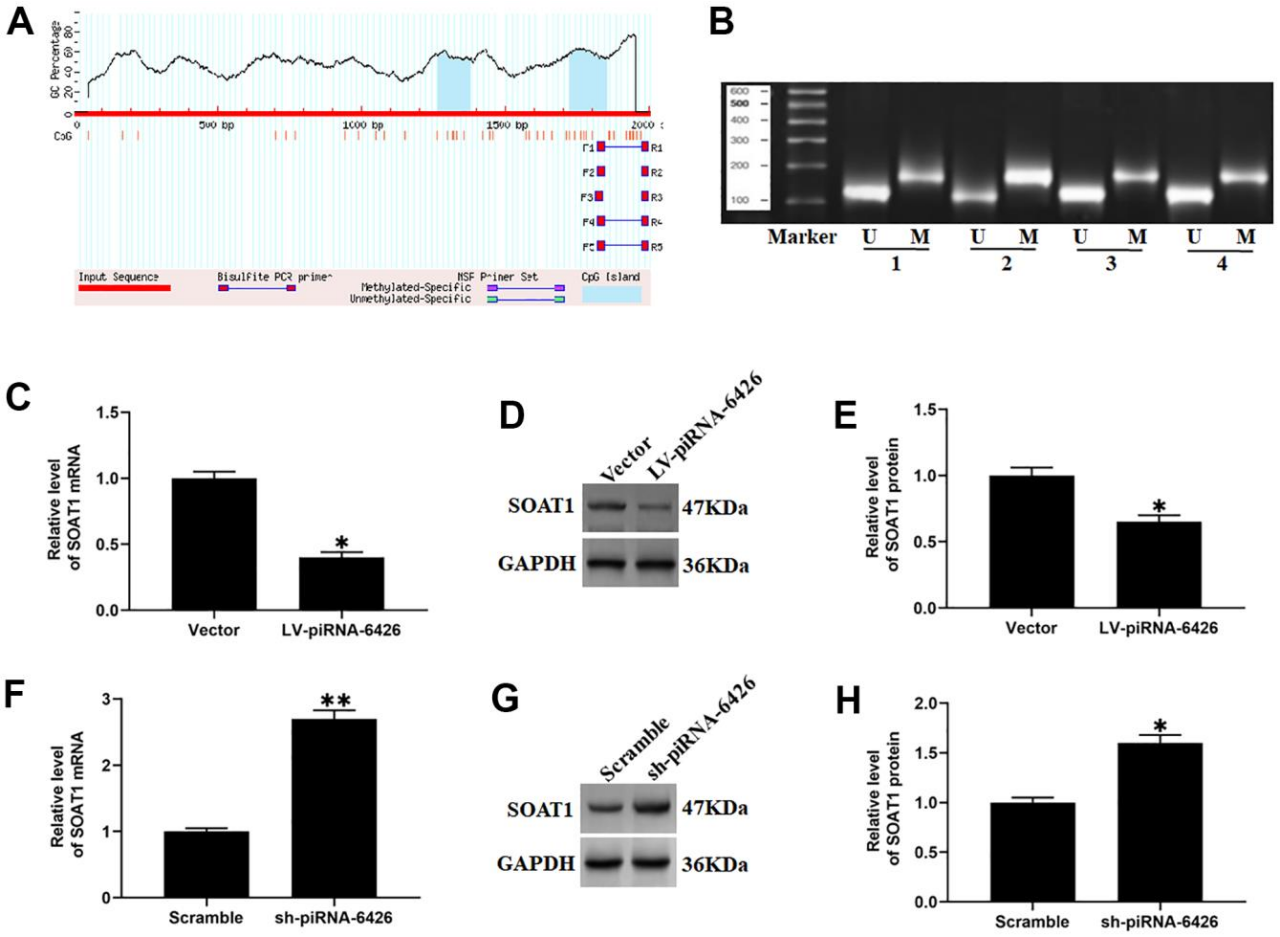
**piRNA-6426 increases the methylation of SOAT1 CpG island.** (**A**) The online prediction software MethPrimer was used to predict the location of the CpG island in the SOAT1 promoter region. (**B**) In a 50 μL system, the extracted DNA (2~5 μg) was denatured with NaOH (final concentration value 0.2 mol/L) at 37° C for 10 min, and 30 μL of just prepared 10 mmol/L hydroquinone and 520/1 40.5% sodium bisulfite were added and mixed, and then paraffin oil was added to isolate from the air, and incubated for 16 h in the dark. The modified DNA was passed through a Wizard DNA purification column (Chemicon) and eluted at room temperature, and then modified by using NaOH (The final concentration is 0.3 mol/L) for 5 min and precipitated by using ethanol, the DNA is dissolved in 20 μL of water, and specific primers were used for methylation-specific PCR (MSP) detection. (**C**) RT-qPCR assay was used to detect the expression of SOAT1 mRNA in cardiomyocytes incubated with LV-piRNA-6426 or empty vector. (**D**, **E**) Western blotting was used to detect the expression level of SOAT1 protein after piRNA-6426 overexpressed. (**F**) RT-qPCR assay was used to detect the expression of SOAT1 mRNA in cardiomyocytes incubated with sh-piRNA-6426 or scrambled shRNA. (**G**, **H**) Western blotting was used to detect the expression level of SOAT1 protein after piRNA-6426 interfered. Values were expressed as mean ± SEM. *P<0.05, n=6.

A study reported that the methylation level SOAT1 is lower in patients with heart disease than normal [16]. We used the online prediction software MethPrimer (http://www.urogene.org/methprimer) to predict the CPG islands in SOAT1 promoter region. The results found that the SOAT1 promoter region contained at least 2 CPG islands (Figure 4A). To investigate the effect of piRNA-6426 on the methylation level of SOAT1 promoter, we overexpressed and interfered with piRNA-6426, and detected the methylation level of SOAT1 promoter and the expression levels of SOAT1 mRNA and SOAT1 protein. To our surprised, compared with the control group, the methylation level of the SOAT1 promoter in the piRNA-6426 overexpression group was significantly increased, and the expression level of SOAT1 mRNA and protein was significantly decreased (Figure 4B–4E). While in piRNA-6426 interference group, the methylation level of SOAT1 promoter was significantly decreased, and the expression levels of SOAT1 mRNA and protein were significantly increased compared with the control group (Figure 4B, 4F–4H).

### piRNA-6426 promotes SOAT1 promoter methylation by recruiting DNMT3B

**Figure 5:**
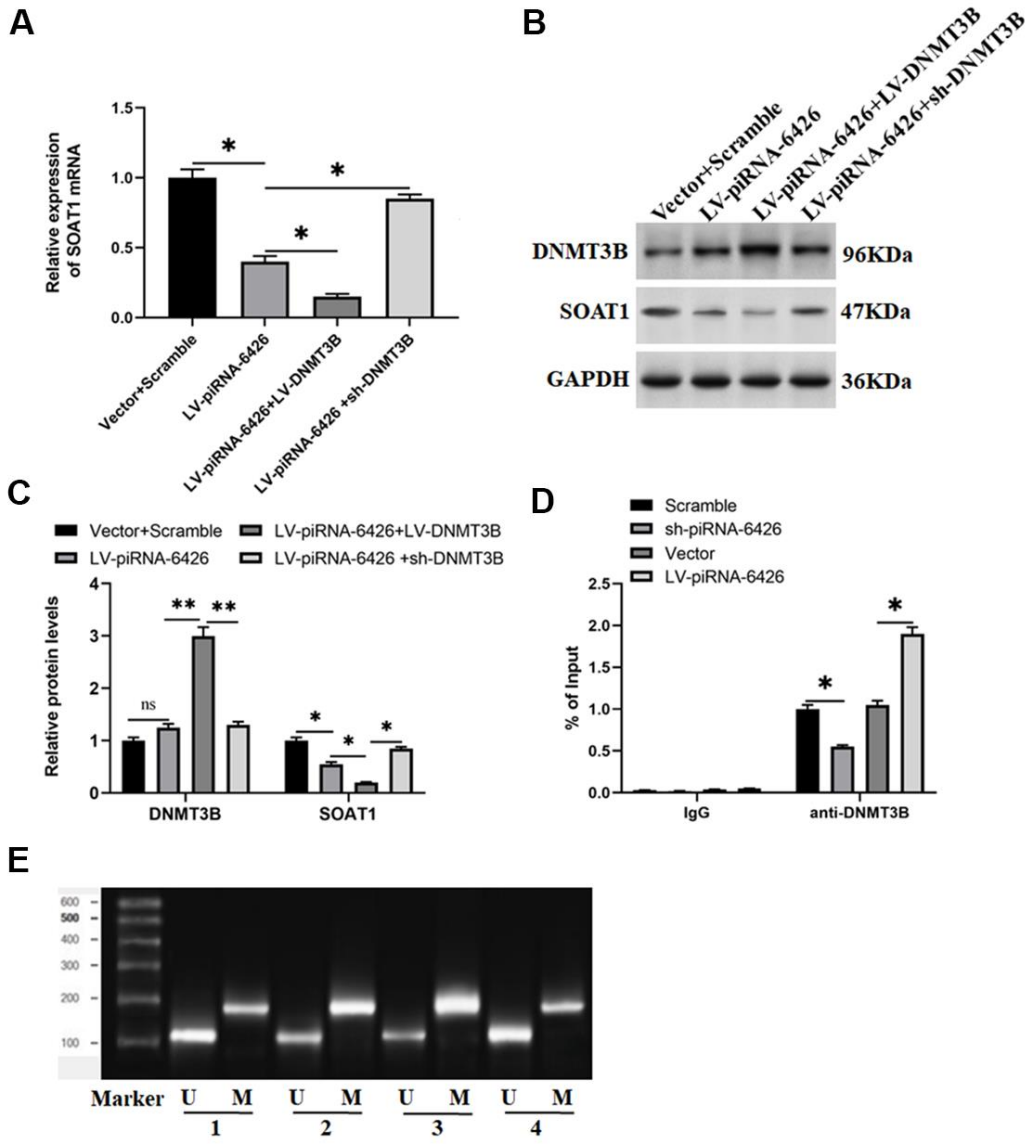
**piRNA-6426 promotes SOAT1 promoter methylation by recruiting DNMT3B.** The cells were transfected with LV-piRNA-6426 or together with LV-DNMT3B or sh-DNMT3B for 48 h. (**A**) RT-qPCR assay was used to detect the expression level of SOAT1 mRNA. (**B**, **C**) Western blotting was used to detect the expression levels of SOAT1 and DNMT3B proteins. (**D**) ChIP-qPCR was used to analyze the enrichment of DNMT3B in the SOAT1 promoter region after piRNA-6426 overexpression or interference, the normal rat IgG (IgG) was used as a negative control, data represent mean values relative to input (% Input). (**E**) MSP assay was used to detect the methylation level of SOAT1 promoter (the groups are consistent with panels **B**, **C**). Values were expressed as mean ± SEM. ns *P*>0.05, *P<0.05, **P<0.01, n=6.

In the current research we found that when piRNA-6426 and DNMT3B were co-overexpressed, the methylation level of SOAT1 was increased and the levels of SOAT1 mRNA and protein were decreased compared with overexpression of piRNA-6426 alone. When piRNA-6426 was overexpressed and DNMT3B was interfered, the methylation level of SOAT1 was decreased and the levels of SOAT1 mRNA and protein were increased compared with overexpression of piRNA-6426 alone (Figure 5A–5C, 5E). Next, we used DNMT3B antibody to perform chromatin immunoprecipitation-quantitative PCR (ChIP-qPCR) on rat cardiomyocytes transfected with piRNA-6426 overexpression vector or piRNA-6426 shRNA. The results showed that the enrichment of DNMT3B in the SOAT1 promoter region of the piRNA-6426 interference group was significantly decreased compared with the control group. While the enrichment of DNMT3B in the SOAT1 promoter region of the piRNA-6426 overexpression group was significantly increased compared with the control group (Figure 5D).

### High methylation level of SOAT1 promoter alleviates hypoxia-induced dysfunction of rat cardiomyocytes

**Figure 6:**
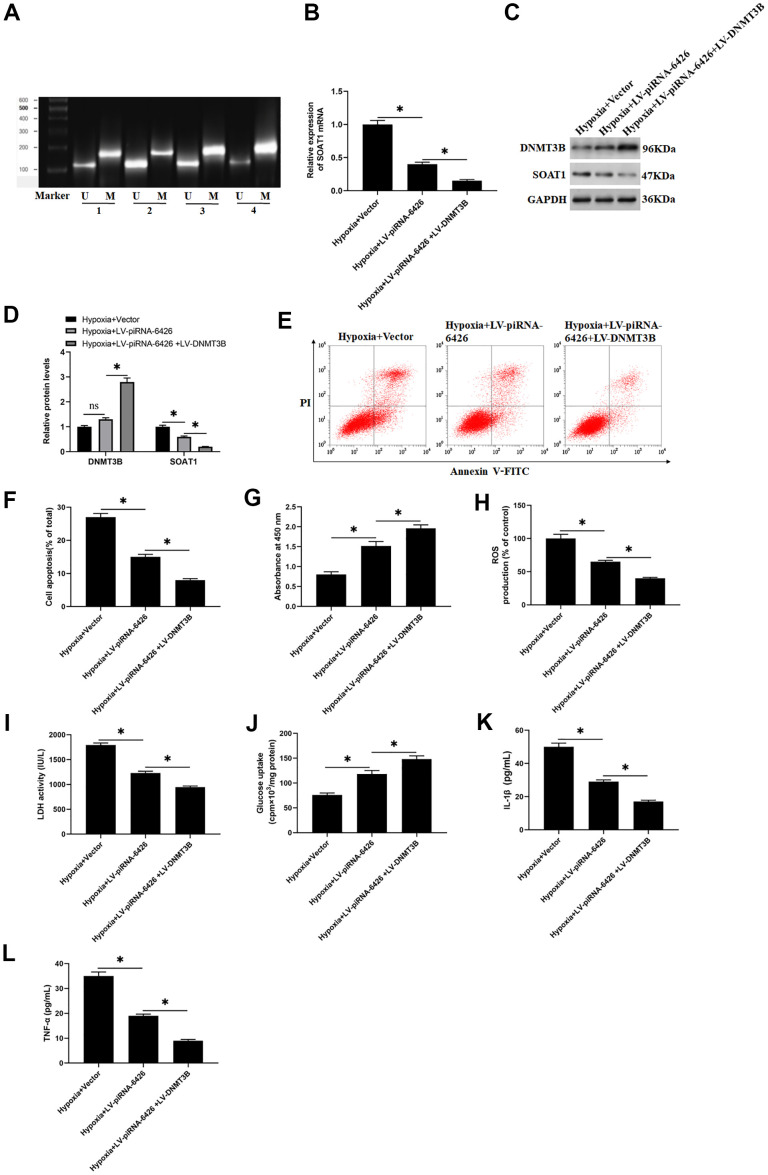
**High methylation level of SOAT1 promoter alleviates hypoxia-induced dysfunction of rat cardiomyocytes.** The cells were induced in a hypoxic incubator for 24 hours to establish a HF cell model after 48 h of per-incubation with LV-piRNA-6426 or together with LV-DNMT3B. (**A**) MSP assay was used to detect the methylation level of SOAT1 promoter. (**B**) RT-qPCR assay was used to detect the expression level of SOAT1 mRNA. (**C**, **D**) Western blotting was used to detect the expression levels of SOAT1 and DNMT3B proteins. (**E**, **F**) Flow cytometry was used to detect cell apoptosis. (**G**) MTT assay was used to identify cell viability. (**H**) The production of ROS was analyzed with DCFH-DA. (**I**) The LDH activity was detected with an ELISA kit. (**J**) D-(2-3H)-glucose uptake assay was used to perform glucose uptake on fully fused rat cardiomyocytes. (**K**, **L**) ELISA kits were used to detect the secretion of IL-1β and TNF-α. Values were expressed as mean ± SEM. ns *P*>0.05, *P<0.05, **P<0.01, n=6.

**Figure 7:**
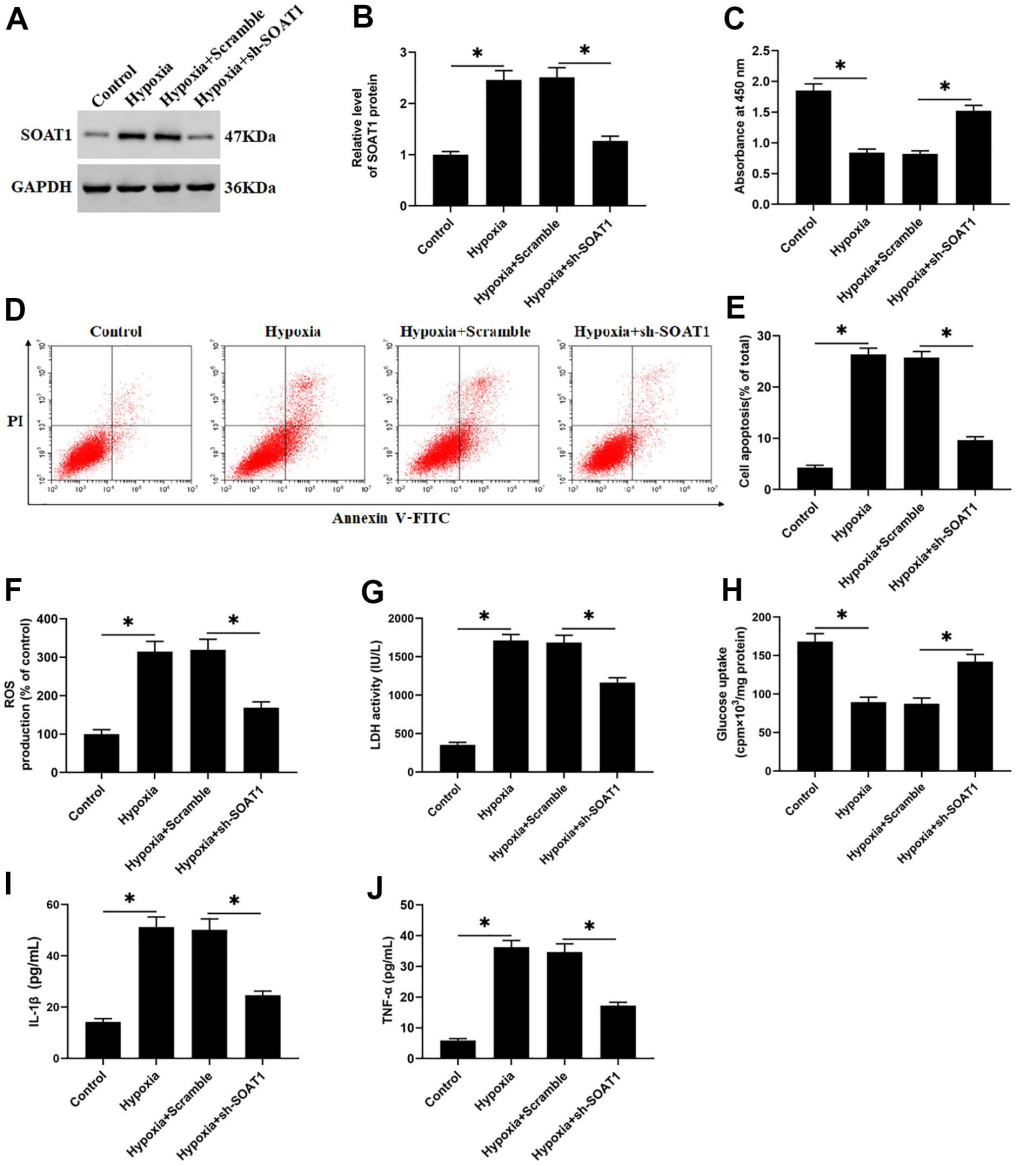
**SOAT1 interference alleviates hypoxia-induced dysfunction of rat cardiomyocytes.** Hypoxia-induced cardiomyocytes were transfected with sh-SOAT1 or negative control scrambled shRNA for 48 h. (**A**, **B**) Western blotting was used to detect the expression level of SOAT1 protein. (**C**) MTT assay was used to test cell viability. (**D**, **E**) Flow cytometry was used to detect cell apoptosis. (**F**) The production of ROS was analyzed with DCFH-DA. (**G**) The LDH activity was detect with a LDH ELISA kit. (**H**) D-(2-3H)-glucose uptake assay was used to perform glucose uptake on fully fused rat cardiomyocytes. (**I**, **J**) ELISA kits were used to detect the secretion of IL-1β and TNF-α. Values were expressed as mean ± SEM. *P<0.05, n=6.

**Figure 8:**
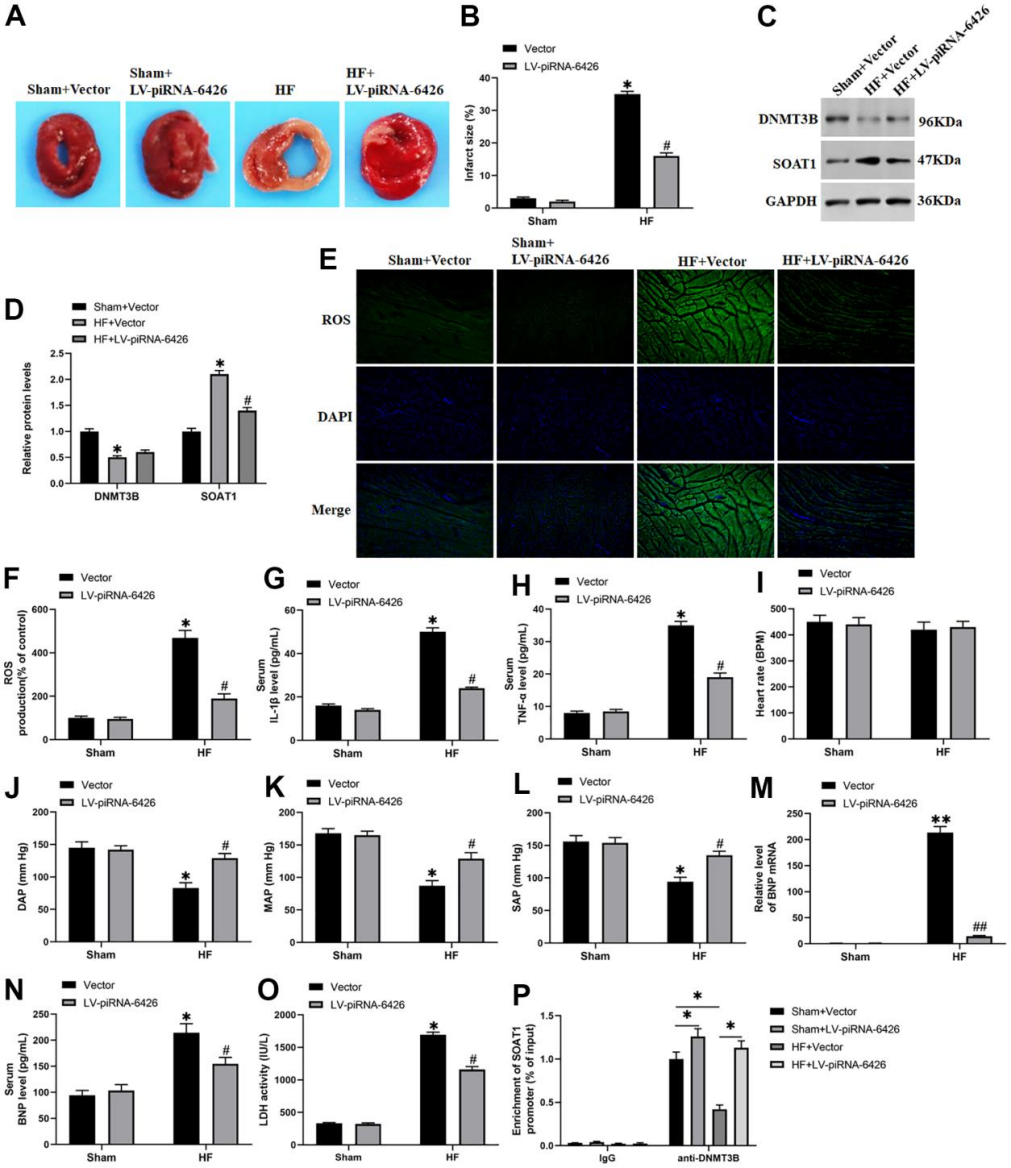
**Overexpression of piRNA-6426 improves cardiomyocyte function in rats with heart failure.** The coronary artery occlusion valve was used to establish a HF rat model. piRNA-6426 gene was cloned into the lentiviral vector. And 8×105 TU lentivirus were injected into the HF mice. The rats in each group were anesthetized after feeding for 4 weeks for the detection of various indicators. (**A**, **B**) TTC staining was used to detect the infarcted area of rat heart. (**C**, **D**) Western blotting was used to detect the expression levels of SOAT1 and DNMT3B proteins. (**E**, **F**) The production of ROS was analyzed with DCFH-DA. (**G**, **H**) ELISA kits were used to detect LDH activity in heart tissue homogenate and the secretion of serum IL-1β content. (**I–L**) The rats were anesthetized by intraperitoneal injection of ketamine (100 mg/kg) and xylazine (10 mg/kg), and then placed on XR900 non-invasive blood pressure monitor (Xinruan, Shanghai, China) to monitor and record the heart rate, diastolic arterial pressure (DAP), mean arterial pressure (MAP) and systolic arterial pressure (SAP). (**M**) RT-qPCR was used to detect the BNP mRNA level in mouse serum. (**N**, **O**) ELISA kits were used to detect the secretion of serum TNF-α and BNP levels. (**P**) The change of DNMT3B bind to the promoter of SOAT1 by chromatin immuno-precipitation (ChIP) method. Values were expressed as mean ± SEM. $ P>0.05, *P<0.05 compared with the sham operation group; ns P>0.05, #P<0.05 compared with the HF group, n=10.

To explore the effect of SOAT1 promoter methylation level on hypoxia-induced rat cardiomyocytes function, hypoxia-induced rat cardiomyocytes were transfected with piRNA-6426 overexpression vector or together with DNMT3B overexpression vector. The results showed that compared with the control group, the methylation level of SOAT1 promoter in the hypoxia-induced group was significantly decreased (Figure 6A). Compared with the hypoxia group, the SOAT1 promoter methylation level, cell viability and glucose uptake in the piRNA-6426 overexpression group were significantly increased (Figure 6A, 6G, 6J), and the levels of SOAT1 mRNA and protein, cell apoptosis, ROS production, LDH activity and the levels of inflammatory factors IL-1β and TNF-α were significantly decreased (Figure 6B–6F, 6H, 6I, 6K, 6L), and DNMT3B overexpression enhanced the effect of piRNA-6426. Also, hypoxia-induced cardiomyocytes were transfected with sh-SOAT1, and the results found that SOAT1 interference decreased the expression level of SOAT1 protein (Figure 7A, 7B), cell apoptosis (Figure 7D, 7E), ROS production (Figure 8F), LDH activity and the levels of inflammatory factors IL-1β and TNF-α after hypoxia induction (Figure 7G, 7I, 7L), and increased the cell viability and glucose uptake (Figure 7C, 7H). The above results suggest that inhibiting SOAT1 expression level could improve hypoxia-induced dysfunction of cardiomyocytes.

### Overexpression of piRNA-6426 improves cardiac function in rats with heart failure

To investigate the effect of piRNA-6426 overexpression on the cardiac function of HF rats, we injected HF rats with lentiviral piRNA-6426 overexpression vector. The results showed that in HF group the cardiac infarction area (Figure 8A, 8B), SOAT1 protein expression level (Figure 8C, 8D), ROS production and LDH activity in rat heart tissue homogenate (Figure 8E, 8F, 8O), BNP mRNA (Figure 8M) and the secretion of serum BNP (Figure 8N), inflammatory factors IL-1β and TNF-α (Figure 8G, 8H) were increased, and the expression level of DNMT3B protein (Figure 8C, 8D) in rat heart tissues and the systolic (Figure 8L), diastolic and mean arterial pressure of rats were decreased compared with the sham operation group (Figure 8J, 8K). In piRNA-6426 overexpression group, the cardiac infarction area (Figure 8A, 8B), SOAT1 protein expression level (Figure 8C, 8D), ROS production and LDH activity in heart tissue homogenate (Figure 8E, 8F, 8O), BNP mRNA (Figure 8M) and the secretion of serum BNP (Figure 8N), inflammatory factors IL-1β and TNF-α (Figure 8G, 8H) were decreased, and the expression level of DNMT3B protein did not change significantly (Figure 8C, 8D), and the systolic (Figure 8L), diastolic and mean arterial pressure of rats were increased compared with the HF group (Figure 8J, 8K). But the heart rate had never changed significantly (Figure 8I). These results demonstrated that piRNA-6426 could alleviate the cardiac dysfunction in HF rats. Next, we studied the association between DNMT3B and SOAT1 gene. We found that HF significantly decreased the level of DNMT3B bound to the promoter of SOAT1 compared with the sham+ Vector group. In HF+ LV-piRNA-6426 group, Overexpression of piRNA-6426 by intraperitoneally injection of LV-piRNA-6426 significantly increased the level of DNMT3B bound to the promoter of SOAT1 compared with the HF+ Vector group (Figure 8P).

**Figure 9:**
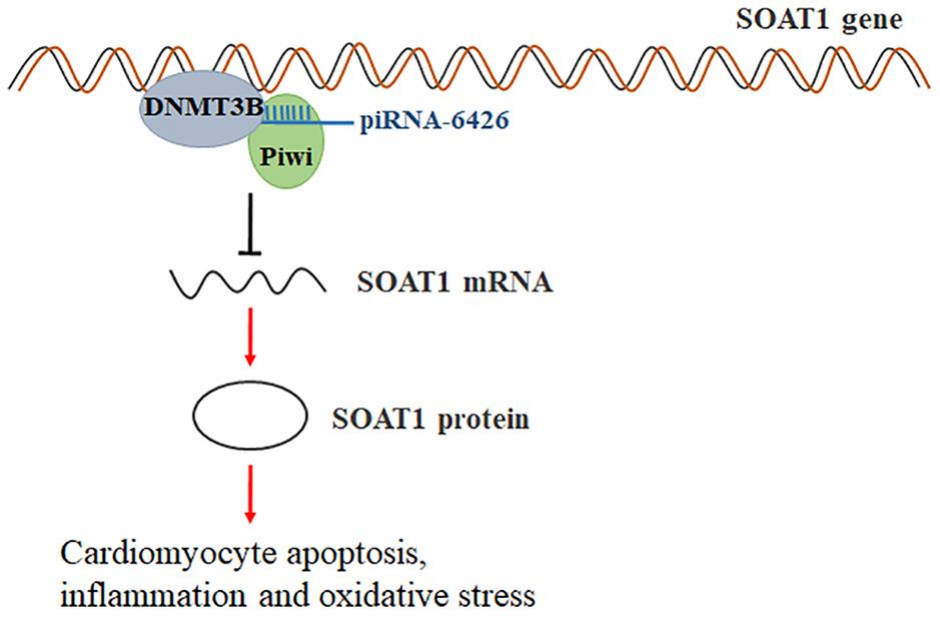
**Schematic of regulation of heart failure by piRNA-6426-mediated methylation of the SOAT1.** piRNA-6426 regulates the methylation level of SOAT1 by recruiting DNMT3B to the promoter of SOAT1, thereby inhibiting cardiomyocytes apoptosis, inflammation and oxidative stress.

Taken together, these findings suggested that piRNA-6426 increases the methylation level of SOAT1 by recruiting DNMT3B to the promoter of SOAT1, thereby inhibiting the cardiomyocyte apoptosis, inflammation and oxidative stress and the progression of HF (Figure 9).

## DISCUSSION

HF is a late clinical symptom of a variety of cardiovascular diseases such as hypertension, diabetes, coronary heart disease and myocardial infarction, its appearance indicates a poor prognosis [23]. In our country, about 1.3% of the 35-year-old population (about 13.7 million people) suffer from the HF [24]. People with HF still face huge challenges in their diagnosis and treatment. piRNA has become a diagnostic marker for more and more diseases, for example, piRNA-54265 is up-regulated in colorectal cancer, and could bind with PIWIL2 protein to form a complex to promote the proliferation and metastasis of cancer cells [25]. piRNA-36712 could inhibit the expression of SEPW1P mRNA by binding with SEPW1P mRNA in breast cancer [26], while piRNA has not been reported in cardiovascular diseases. Related sequencing results showed that the expression of piRNA-6426 was down-regulated in the blood of patients with HF compared with normal people [13], which is consistent with our results. We extracted RNA from the blood of 10 HF patients and 10 volunteers, and used specific primers to detect the level of piRNA-6426. The results showed that the expression of piRNA-6426 in the blood of most patients was down-regulated compared with the normal volunteers. Subsequently, we detected the expression of piRNA-6426 in hypoxia-induced rat cardiomyocytes by using RT-qPCR, and the results found that the expression of piRNA-6426 was down-regulated compared with the control group. While piRNA-6426 overexpression could significantly increase cell viability and glucose uptake in hypoxia-induced cardiomyocytes, and decreased cell apoptosis, the production of ROS, LDH activity and the levels of inflammatory factors IL-1β and TNF-α. Moreover, after injection of piRNA-6426 overexpressing lentiviral vector into HF rats, it showed that inflammation and health index of rat hearts were significantly improved. It suggested that piRNA-6426 may be related to the progression of HF.

At present, more and more studies found that DNA methyltransferase affects the progression of HF by regulating the methylation level of related genes [27, 28]. It is reported that the DNMT3B gene knockout mice have severe myocardial insufficiency and myocardial thinning, developing into HF, suggesting that this may be related to the absence of epigenome modifiers [7]. In addition, in the left ventricular dysfunction of mice caused by diabetes, the expression of DNMT3B could be increased to promote the methylation of the src homology 2 domain-containing transforming protein C1 (p66Shc) promoter and alleviate the cardiac dysfunction [29]. Recent studies found that piRNA could regulate disease progression by forming a complex with methyltransferase [30]. For example, piRNA-823 promotes the progression of multiple myeloma by regulating the activity of DNMT3B [12]. piR-DQ541777 promotes the enrichment of DNA methyltransferase 3A (DNMT3a) in the CDK5 regulatory subunit associated protein 1 (cdk5rap1) promoter region by bound to DNMT3a, which regulated the methylation level of the cdk5rap1 promoter and aggravated the neuropathic pain caused by chronic constriction injury (CCI) of the sciatic nerve in mice [22]. In this study, we verified the binding of piRNA-6426 and DNMT3B in rat cardiomyocytes with RIP assay. And we found that piRNA-6426 overexpression or interference had no prominent effect on the expression level of DNMT3B protein in cardiomyocytes.

A study showed that the methylation level of SOAT1 in patients with coronary heart disease is significantly decreased [16]. We found that the overexpression of piRNA-6426 could increase the methylation level of SOAT1 promoter and reduce the expression levels of SOAT1 mRNA and protein, while the interference of piRNA-6426 had the opposite effect. And the results of ChIP assay showed that after interfering with piRNA-6426, the enrichment of DNMT3B in the SOAT1 promoter region was significantly decreased, while the overexpression of piRNA-6426 had the opposite effect. And co-overexpression DNMT3B enhanced the promotion of piRNA-6426 overexpression on the methylation level of SOAT1 promoter and inhibition in levels of SOAT1 mRNA and protein, while DNMT3B interference reversed this promotion effect. Simultaneously, increasing the methylation level of SOAT1 promoter or SOAT1 interference could effectively alleviate the hypoxia-induced dysfunction of rat cardiomyocytes. Based on the above results, we determined that overexpression of piRNA-6426 promoted the enrichment of DNMT3B in the SOAT1 promoter region, thereby increasing the methylation level of SOAT1 promoter and alleviating HF cell dysfunction and HF rat heart function.

In this study, we demonstrated that piRNA-6426 alleviates HF by promoting DNMT3B-mediated SOAT1 methylation. piRNA-6426 is expected to become a new target for HF diagnosis and treatment.

## CONCLUSIONS

Together, the present data suggest that the expression of piRNA-6426 in the peripheral blood of HF patients was significantly lower than volunteers. And piRNA-6426 alleviates hypoxia-induced cardiomyocyte dysfunction and heart failure in rats by regulating DNMT3B-mediated methylation of SOAT1 promoter, it provides a new direction for the treatment of HF.

## MATERIALS AND METHODS

### Human blood samples

In this study, we collected blood from 25 volunteers and 25 heart failure patients from the anterior elbow vein, each with 5 mL of blood, and analyzed the correlation between the gene expression values obtained by RT-qPCR. This experiment was approved by the ethics committee of Shaanxi Provincial People’s Hospital. Each patient or volunteer gave informed consent and signed a written informed consent.

### Isolation and culture of cardiomyocytes

The hearts of SD rats aged 1 to 3 days were dissected, and the ventricles were cut into small pieces. The tissues were transferred to HEPES-buffered saline containing 0.1% collagenase type IV, 0.1% trypsin, 15 μg/mL DNase I and 1% fetal bovine serum, and incubated at 37° C for 15 minutes. After digestion, culture medium containing 10% fetal bovine serum was added to neutralize trypsin. After centrifugation, the dissociated cells were re-suspended in Dulbecco's modified Eagle's medium (DMEM, Gibco, Rockville, MD, USA) containing with 10% (v/v) fetal bovine serum (FBS, IBM, Almon, NY, USA), 100 U/mL penicillin (Millipore, Boston, MA, USA) and 100 U/mL streptomycin (Millipore) for 24-36 h at 37° C with 5% CO_2_ in a humidity incubator. Then cardiomyocytes were cultured in hypoxia incubator with 94% N_2_, 5 % CO_2_ and 1% O_2_ for 24 h to induce injury. In the study, rat cardiomyocytes under normoxia were treated as controls.

### Lentivirus production and transfection

Lentivirus overexpression vector containing piRNA-6426 (LV-piRNA-6426) and DNMT3B (LV-DNMT3B) and its negative control were constructed by Sangon, Shanghai, China. In brief, based on the gene sequence of piRNA-6426 and DNMT3B which were recorded in gene bank of national center for biotechnology information (NCBI), and design principle of short hairpin RNAs (shRNAs). The ds oligonucleotides were ligated into RNAi-Ready pSIREN-DNR-DsRed-Express Vector (Clontech, Mountain View, CA, USA). Then the Donor Vector was inserted into LV-MAX Vector (Clontech) to be constructed as the recombinant pLP-LV-MAX piRNA-6426 shRNA (LV-sh-piRNA-6426). The LV vector with a scrambled shRNA was prepared as control (Scramble). And LV-sh-DNMT3B was synthesized with the same method. All transfections were performed by using Lipofectamine® 3000 (Thermo, Waltham, MA, USA) according to the manufacturer's instructions.

### Heart failure rat model

This study was conducted in accordance with the "Guidelines for the Care and Use of Laboratory Animals", and the animal experiments were approved by the Shaanxi Provincial People’s Hospital Animal Research Committee. Forty male Sprague-Dawley (SD) rats (weighing 250-300 g) about 2 months old were purchased from Experimental Animal Center of Xi'an Jiaotong University Medical College. The rats were divided into: sham operation + injection of empty vector group, sham operation + injection of piRNA-6426 overexpression vector group, heart failure + injection of empty vector group, heart failure + injection of piRNA-6426 overexpression vector group. The rats were anesthetized by intraperitoneal injection of ketamine (100 mg/kg) and xylazine (10 mg/kg) before operation. During the operation, the body temperature of rats was maintained at 37.5° C through a thermostatic control panel. In the study of dysfunction, coronary artery occlusion was used to establish a HF model, which caused ischemic myocardial damage. In the sham operation group (sham), the thoracic cavity of rats was only opened and sutured, and the rats were intraperitoneally injected with 8×10^5^ TU lentivirus which were inserted or not inserted with piRNA-6426 sequence. In the HF group, rats were intraperitoneally injected with 500 μL of 8×10^5^ TU lentivirus which were inserted or not inserted with piRNA-6426 sequence. Four weeks after the operation, the rats were euthanized by injecting 150 mg/Kg pentobarbital sodium for cardiac isolation after blood pressure measurements and venous blood collection. TTC (2,3,5-triphenyltetrazolium chloride) staining was performed to detect the area of myocardial infarction, and the serum and heart tissues were collected for the next experiment.

### Processing for heart tissue homogenate

The hearts were collected, washed with cold phosphate buffered saline (PBS), quick-frozen with liquid nitrogen, and then ground into fine powder. 30-40 mg of myocardial tissues were taken from each group and homogenized by using Brinkmann homogenizer with Kinematica 87 (IBM, Almon, NY, USA) in homogenization buffer (Gibco, Rockville, MD, USA) for 3-5 s. The homogenization procedure was carried out at 4° C. The samples were placed on a rotator (Bio-Rad, Hercules, CA, USA) and mixed for at least 15 minutes to warm the suspension to room temperature before being used in subsequent experiments.

### Glucose uptake assay

D-(2-^3^H)-glucose uptake was used to perform glucose uptake experiments on fully fused rat cardiomyocytes cultured in 96-well plates. The cells were exposed to 20 mM and 200 mM METH for 24 h. Then cells were incubated overnight in glucose-free DMEM media containing equimolar of D-(2-^3^H)-glucose (1.0 mCi) and non-radiolabeled glucose. After washing off the excess ^3^H-glucose with Krebs-Ringer phosphate-HEPES (KRPH) buffer (Sangon), cellular protein was precipitated with 10% trichloroacetic acid (TCA, Millipore, Boston, MA, USA) at 4° C for 15 min. Precipitated proteins were transferred onto a 96 well nitrocellulose filter using the Unifilter-96 well Harvester as the manufacturer’s instructions. Using a Beckman 96 well plate reader, radioactivity was measured by Beckman 96 well plate reader and β-top counter (Roche, Basel, Switzerland).

### Detection of reactive oxygen species (ROS) level

The 2',7'-dichlorofluorescein diacetate (DCFH-DA) method was used to determine the level of ROS. Briefly, at the end of the experiment, the cells were washed with PBS and incubated with DCFH-DA (Millipore, Boston, MA, USA) at a final concentration of 10 mM at 37° C in the dark for 1 h. After washing the cells twice, using PBS to remove the extracellular DCFH-DA, the fluorescence intensity was measured with a flow cytometer (Becton Dickinson, San Diego, CA, USA), the excitation wavelength was 488 nm, and the emission wavelength was 525 nm. The level of intracellular ROS was expressed as a percentage of the control.

### Detection of LDH concentration

The serum of rats and supernatant of broken cardiomyocytes were collected. The concentration of lactate dehydrogenase (LDH) was determined with an ELISA kit (Invitrogen, Carlsbad, CA, USA). The absorbance values of all samples at 450 nm were determined and the LDH concentration was calculated using the standard curve.

### CCK-8 assay

The Cell Counting Kit-8 (CCK-8) assay (Sigma-Aldrich, St. Louis, MO, USA) was performed to detect cardiomyocyte proliferation. In brief, cardiomyocytes were seeded in a 96-well plate at a density of 5×10^3^ cells/well. After culture and treatment, cells were washed with cold PBS, and 10 μL of CCK-8 solution was added to each well and incubated with cardiomyocytes for 2 h at 37° C in a humidified atmosphere with 5% CO_2_. The absorbance was measured at 450 nm with a microplate reader (Bio-Rad, Hercules, CA, USA).

### Cell apoptosis

We used FITC-Annexin Apoptosis Detection Kit (Invitrogen, Carlsbad, CA, USA) to detect the level of apoptosis in each group according to the instructions. In brief, cardiomyocytes of different groups were seeded in 6-well plates, after culture and treatment, the cells were harvested and washed twice with cold PBS. 1× binding buffer was used for cell recovery, and then the cells were incubated in the dark with Annexin V/PI solution for about 20 min at room temperature. Flow cytometry system (Becton Dickinson, San Diego, CA, USA) was used to analyze the apoptosis rate of cardiomyocytes.

### RT-qPCR

Trizol reagent (Invitrogen) was used to extract total RNA from human serum, rat myocardial tissues and cardiomyocytes according to the manufacturer's instructions. NanoDrop ND-1000 (NanoDrop Technologies) was used to determine the concentration of purified RNA samples. For piRNAs, 1 μg of total RNA was reverse-transcribed to cDNA using the miRNA 1st Strand cDNA Synthesis Kit (by stem-loop) (Vazyme, Jiangsu, China) and a stem-loop RT primer (Genscript, Jiangsu, China). For mRNAs, 1 μg of total RNA was reversely-transcribed to cDNA using SuperScript IV reverse transcriptase (Thermo Fisher Scientific, Waltham, MA, USA). Finally, 1 μL of cDNA was applied in RT-qPCR analysis using SuperScript IV reverse transcriptase (Thermo Fisher Scientific, Waltham, MA, USA). Real-time quantitative PCR analyses were conducted with the Platinum™ Taq DNA Polymerase High Fidelity (Thermo Fisher Scientific) with Applied Biosystems 7500 Real-Time PCR System (Applied Biosystems, Foster City, CA, USA) following the thermal cycling procedures, 95° C for 1 min, 30 cycles of 95° C for 30 s, 60° C for 1 min and 72° C for 1 min. The PCR reaction system contained 12.5 μL of Taq DNA Polymerase High Fidelity, 1.0 μL of RT primer, 1 μL of cDNA sample, and double distilled H_2_O was used to make up the vacant volume. The primer sequences used in this study were as follows: piRNA-6426 RT: 5'-CTC AAC TGG TGT CGT GGA GTC GGC AAT TCA GTT GAG TAG GAC TT-3'. piRNA-6426 S: 5'-GCC GAG AGG AGT GAA GTC TAC-3'; AS: 5'-TCA ACT GGT GTC GTG GAG TCG-3'. SOAT1 S: 5'-AGC CCA GAA AAA TTT CAT GGA CAC ATA CAG-3'; AS: 5'-CCC TTG TTC TGG AGG TGC TCT CAG ATC TTT-3'. Brain Natriuretic Peptide (BNP): 5'-TCT CCA GAG CAA TTC AAG AT-3'; AS: 5'-AAC AAC TTC AGT GCG TTA CA-3'. GADPH S: 5'-GAG TCA ACG GAT TTG GTC GT-3'; AS: 5'-AGC ACT GTG TTG GCG TAC AG-3'. U6 S: 5'-GTG CTC GCT TCG GCA GCA CAT ATA C-3'; AS: 5'-AAA AAT ATG GAA CGC TTC ACG AAT TTG-3'. Gene expression was normalized to Glyceraldehyde-3-phosphate dehydrogenase (GAPDH), and piRNA-6426 expression was normalized to U6 using the 2^−ΔΔCt^ method.

### Methylation-specific PCR analysis

Genomic DNA was extracted from the control group or IPTG-treated cells with QIAamp DNA Mini Kit (Qiagen, Dusseldorf, Germany). The DNA was modified with sodium bisulfite and analyzed according to the procedure of the CpGenome DNA Modification Kit (Chemicon, Springfield, IL, USA). The primers were methylated sense: 5'-TAT ATT TTA TTG TTG GGG TGG AAG T-3' and antisense: 5'-TAA TAA TCC CCA AAT TAC CAA ACT C-3' for the methylated sequence of human SOAT1 promoter and unmethylated sense: 5'-TGT GCA GAT AAG CCA GCG AT-3' and antisense: 5'-TCC TAG GCC GAC TGG AAG AA-3' for the unmethylated sequence of human SOAT1 promoter. The predicted products for methylated and unmethylated DNA are 179 and 116 bp. The DNA were amplified in a 50 μL of reaction system and the mixture contains 5 μL of 10×PCR buffer, 15 μL of 25 mmol/L MgCl_2_, 2.5 μL of 25 mmol/L deoxynucleotide triphosphate, and 1 μL of each primer (300 ng/μL) and 0.5 units of DNA polymerase (Bio-Rad, Hercules, CA, USA). PCR was performed in a thermal cycler for 35 cycles (denaturation at 95° C for 1 min, annealing at 56° C for 2 min, and extension at 72° C for 1 min), followed by extension at 72° C for 10 min. The PCR products were separated in a 2% agarose gel, stained with ethidium bromide, and visualized under UV illumination.

### Western blotting

To measure the protein expression level, the myocardial tissues and the cardiomyocytes were lysed by using RIPA lysis buffer (Thermo Fisher Scientific, Waltham, MA, USA). BCA protein assay kit (Thermo Fisher Scientific) was used to detect the protein concentration. Then, equal amounts of proteins (30 μg/lane) were separated by 10% SDS-PAGE and electro-transferred onto PVDF membranes (Thermo Fisher Scientific). Membranes were incubated with 5% non-fat milk for 2 h at room temperature, and then incubated with primary antibodies which were purchased from Abcam (Cambridge, UK) including rabbit monoclonal anti-DNMT3B (1:1000, ab79822), rabbit polyclonal anti-SOAT1 (1:1000, ab39327) and anti-Histone H3(1:2500, ab1791) and mouse monoclonal anti-GADPH (1:2000, ab9485) overnight at 4° C. And then the membranes were incubated with secondary antibody horseradish peroxidase (HRP)-conjugated goat anti-rat IgG (1:200, Covance, SMI-5040C) for 2 h, and GADPH was used as a loading control. The expression level of protein was analyzed by chemiluminescence and quantified using ImageJ software (National Institutes of Health, Bethesda, MA, USA) and E-Gel Imager (Thermo Fisher Scientific).

### Enzyme-linked immunosorbent assay (ELISA)

The lysed cardiomyocytes and serum were collected, and the impurities were removed through a 0.45-μm filter to detect inflammatory factors. The secretion levels of IL-1β, TNF-α and BNP were determined with specific enzyme-linked immunosorbent assay (ELISA) kits (Takara Biotechnology, Dalian, China) according to the manufacturer's instructions.

### Chromatin immunoprecipitation assay

Chromatin immunoprecipitation (ChIP) detection was performed by using Enzymatic Chromatin IP kit (Invitrogen, Carlsbad, CA, USA) according to the manufacturer's instructions. Briefly, the cardiomyocyte lysate was cross-linked with 1% formaldehyde, and then a protease inhibitor ChIP lysis buffer was added. Ultrasound was used to disrupt the lysate to obtain chromatin with an average size of 200-500 bp. Also, rat heart tissues were crosslinked with 1% formaldehyde followed by adding the ChIP lysis buffer with protease inhibitors. Sonicating the tissue to obtain chromatin with an average size of 200-500 bp. And then the immunoprecipitation was incubated with anti-DNMT3B antibody and normal rat IgG overnight at 4° C. Immune complexes were collected with protein G Agarose Beads (Cell Signaling Technology, Boston, MA, USA) after pre-incubation with salmon sperm DNA and BSA for 1 h at 4° C. The beads were washed and eluted with elution buffer. The elution was incubated at 65° C for 2 h to reverse the cross-linking after adjusting the NaCl concentration. The DNA was purified with a Thermo Scientific GeneJET Viral DNA kit (Thermo Fisher Scientific), and amplified with the Platinum™ Taq DNA Polymerase High Fidelity, and quantified with Applied Biosystems 7500 Real-Time PCR System.

### RNA immunoprecipitation (RIP)

RNA was isolated from cardiomyocytes with the RNA-Binding Protein Immunoprecipitation (RIP) Kit (Sigma-Aldrich, St. Louis, MO, USA) according to the manufacturer’s protocol. Cardiomyocytes were lysed in complete RIP lysis buffer. 10 μL of the supernatant of RIP lysate were removed and place into a new tube that was labeled “input”. This input sample was stored at -80° C until RNA purification. 5 μg of the antibody (anti-DNMT3B or normal rat IgG) were added to the tube and incubated rotationally for 30 min at room temperature. The beads were washed twice with cold RIP Wash Buffer. All the tubes contained cell lysate, antibodies and beads were incubated rotationally for 4 h overnight at 4° C. After the beads were washed, 20 μL of the beads were removed and placed into a new tube for Western blot analysis. Then, all tubes were incubated with proteinase K at 55° C for 30 min. The supernatant was transferred into a new tube. Finally, the RNA was extracted and analyzed by RT-qPCR.

### TTC staining

The rats were euthanized by injecting 150 mg/Kg pentobarbital sodium, before the hearts were removed and stained with 2,3,5-triphenyltetrazolium (TTC) staining. Briefly, the hearts were isolated and cut into 4 mm slices, which were immediately immersed in 1% TTC solution (BioTeke, Beijing, China) in phosphate buffer (pH 7.4) at 37° C for 15 min. After TTC staining, the sections were fixed with 10% formaldehyde. The infarct area was white, while the normal area was red. The sections were photographed with a digital camera, and Image-Pro Plus 6.0 was used for area statistical analysis to calculate the left ventricular area (the total myocardium) and the area of the infarct area (white). The ratio of infarct area to left ventricular area was infarct size (expressed as a percentage).

### Statistical analysis

Each experiment was repeated at least three times and results were presented as means ± standard error of means (SEM) after being analyzed by using SPSS 22.0 (SPSS Inc., Chicago, IL, USA), Student's t test was used to estimate the significance of differences between two unpaired samples, and ANOVA with Tukey’s post hoc test was used to compare differences among groups. *P* < 0.05 was considered statistically significant.

### Ethics approval and consent to participate

All patients gave informed consent and signed an informed consent form. All samples obtained in this study were approved by the ethics committee of the Shaanxi Provincial People’s Hospital and abided by the ethical guidelines of the Declaration of Helsinki. All animal experiments comply with the guidelines for the care and use of laboratory animals established by the National Institutes of Health (Bethesda, MD, USA).

### Availability of data and materials

The datasets used during the present study are available from the corresponding author on reasonable request.
